# Induced pluripotent stem cells as a model for diabetes investigation

**DOI:** 10.1038/srep08597

**Published:** 2015-02-26

**Authors:** J. Stepniewski, N. Kachamakova-Trojanowska, D. Ogrocki, M. Szopa, M. Matlok, M. Beilharz, G. Dyduch, M. T. Malecki, A. Jozkowicz, J. Dulak

**Affiliations:** 1 Department of Medical Biotechnology, Faculty of Biochemistry, Biophysics and Biotechnology, Jagiellonian University, Krakow, Poland; 2 Clinic of Metabolic Diseases, Jagiellonian University Medical College, Krakow, Poland; 3 University Hospital Krakow Poland; 4 II Clinic of General Surgery, Collegium Medicum, Jagiellonian University, Krakow, Poland; 5 Department of Clinical and Experimental Pathomorphology, Jagiellonian University Medical College, Krakow, Poland; 6 Malopolska Centre of Biotechnology, Jagiellonian University, Poland

## Abstract

Mouse and human induced pluripotent stem cells (iPSCs) may represent a novel approach for modeling diabetes. Taking this into consideration, the aim of this study was to generate and evaluate differentiation potential of iPSCs from *lep^db/db^* (db/db) mice, the model of diabetes type 2 as well as from patients with Maturity Onset Diabetes of the Young 3 (HNF1A MODY). Murine iPSC colonies from both wild type and db/db mice were positive for markers of pluripotency: Oct3/4A, Nanog, SSEA1, CDy1 and alkaline phosphatase and differentiated *in vitro* and *in vivo* into cells originating from three germ layers. However, our results suggest impaired differentiation of db/db cells into endothelial progenitor-like cells expressing CD34 and Tie2 markers and their reduced angiogenic potential. Human control and HNF1A MODY reprogrammed cells also expressed pluripotency markers: OCT3/4A, SSEA4, TRA-1–60, TRA-1-81, formed embryoid bodies (EBs) and differentiated into cells of three germ layers. Additionally, insulin expressing cells were obtained from those partially reprogrammed cells with direct as well as EB-mediated differentiation method. Our findings indicate that disease-specific iPSCs may help to better understand the mechanisms responsible for defective insulin production or vascular dysfunction upon differentiation toward cell types affected by diabetes.

Diabetes mellitus is a group of complex metabolic diseases affecting more than 300 million people worldwide^1^. The most common form is type 2 diabetes (T2D) which is characterized by the lack of proper response to insulin in the peripheral tissues, a phenomenon known as the insulin resistance^2^. Additionally, T2D is accompanied by relative insulin deficiency resulting from defects in the secretion of this hormone^3^. Among others, this disease is also closely associated with cardiovascular complications, with endothelial cells being significantly affected^4^. *Lep^db/db^* is a mouse strain frequently utilized as T2D model with the mutation in leptin receptor resulting in hyperphagy, obesity, hyperinsulinemia and hyperglycemia, the latter three reflecting clinical features of T2D patients^5^,^6^. Importantly, animal models based on disturbed leptin activity are often used to test new therapeutic approaches against T2D^7^,^8^.

In contrast to T2D, monogenic forms of diabetes are rather rare, with the maturity onset of diabetes of the young (MODY) being the most frequent among them, constituting 1–2% of diabetic cases^9^. HNF1A MODY (MODY3) is the most common form which results from the mutations in hepatocyte nuclear factor 1 alpha (*HNF1A*) gene^10^. This nuclear receptor is expressed in several organs, including pancreas, and controls the production of multiple factors responsible for glucose uptake, glycolysis and mitochondrial metabolism. Patients with MODY3 usually suffer from postprandial hyperglycemia and require pharmacological treatment with sulphonylureas or, in later stage, with insulin^11^. The molecular basis of this disease, similarly to T2D, has been characterized with the application of cell lines and animal models^12^, however utilization of patient-specific biological material could significantly enlarge our knowledge on the mechanisms of defective insulin production by the pancreatic β cells.

Induced pluripotent stem cells (iPSCs) which resemble embryonic stem cells with their infinite self-renewal capacity as well as the ability to differentiate into all cell types constituting adult organism^13^,^14^, may provide the new possible source of such patient-specific material. Indeed, many groups demonstrated a feasibility of generating human iPSCs from patients suffering from different illnesses including metabolic disorders like type 1 and 2 of diabetes^15^,^16^ as wells as five forms of MODYs^17^,^18^. Some of these studies described differentiation of hiPSCs towards insulin-producing cells^18^, however further optimization of this process seems to be necessary, especially for different types of MODYs. Additionally, iPSCs have been obtained from murine model of type 1 diabetes^19^ but not the others.

Here we report successful generation of induced pluripotent stem cells from the *Lep^db/db^* mice as the animal model of T2D as well as reprogrammed cells from patients diagnosed with HNF1A MODY. Additionally, this study demonstrates the feasibility of production of such cells to investigate the effects of diabetes on different cell types. Generated iPSCs can be further utilized to analyze the molecular mechanisms responsible for pathophysiology of diabetic disorders or be used for drug screening.

## Results

### Generation and characterization of mouse db/db iPSCs

**Figure 1:**
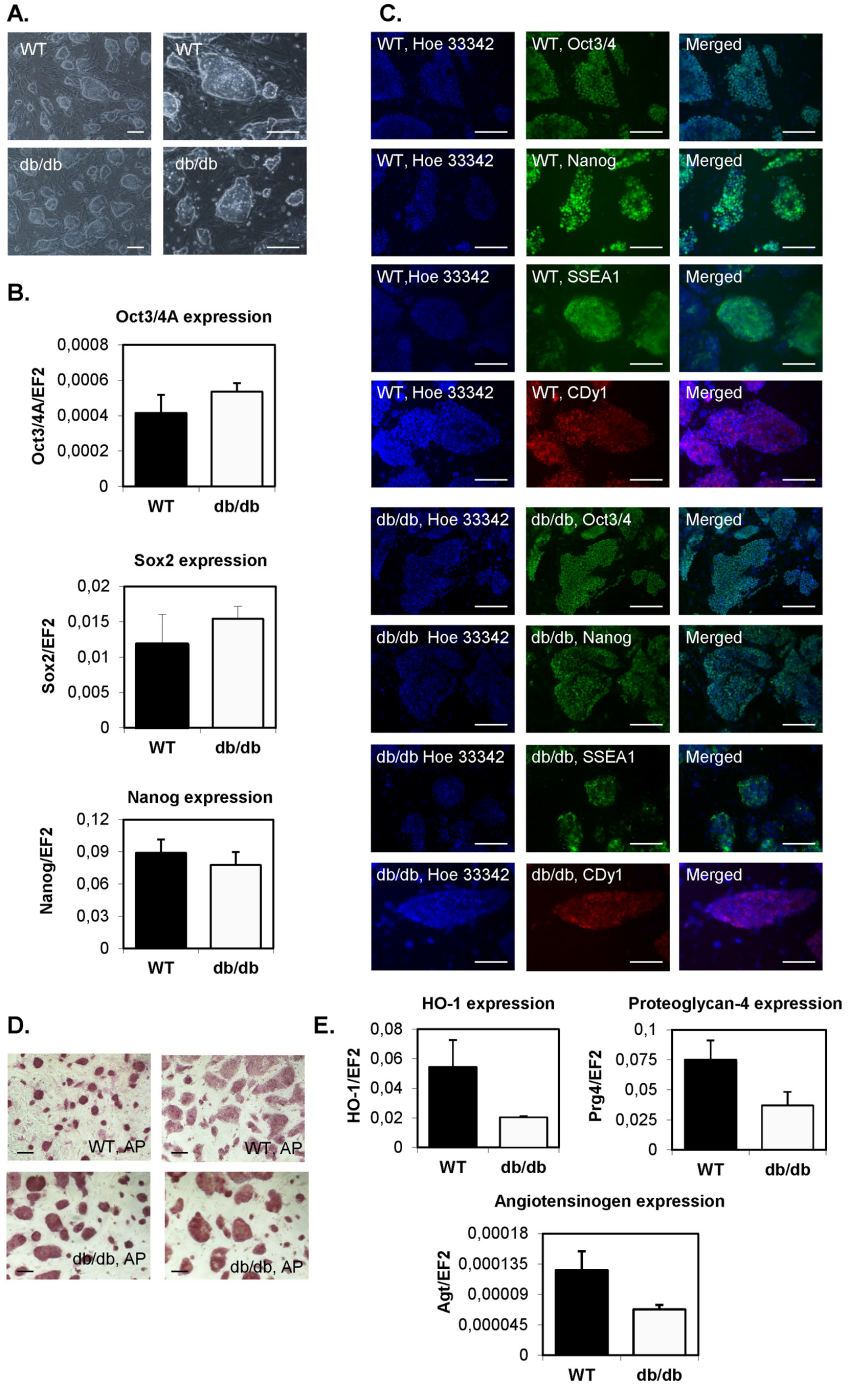
Generation of wild type (WT) and *Lep^db/db^* (db/db) iPS cells. (A). Morphology of WT and db/db iPS cells. Representative pictures (scale bar – 100 μm). (B). Expression of pluripotency markers: Oct3/4A, Nanog and Sox2 in control and db/db iPSCs. Quantitative RT-PCR analysis. N = 3. (C). Immunofluorescence staining of pluripotency markers: Oct3/4A, Nanog, SSEA1 and CDy1 in WT and db/db iPS cells (scale bar – 100 μm). (D). Expression of alkaline phosphatase in WT and db/db iPS cells. Representative pictures (scale bar – 100 μm). (E). Expression of genes associated with endothelial lineage development: HO-1, proteoglycan-4 and angiotensinogen. Quantitative RT-PCR analysis. N = 2. EF2 gene was used as housekeeping gene. Each bar represents mean + SEM. The numerical data presented in Fig. 1B and 1E were not statistically significant.

Transduction of tail tip fibroblasts isolated from control wild type (WT) and *Lep^db/db^* mice with STEMCCA lentiviral vectors resulted in generation of multiple colonies (within 2–3 weeks of reprogramming process) resembling mouse embryonic stem cells which could be further picked up and expanded (Figure 1A). No differences between cells from WT and *Lep^db/db^* mice were observed. This indicates that leptin signaling, which is disturbed in db/db cells is not required for proper reprogramming outcome. Additionally, no changes in morphology of db/db iPSCs were noticed in comparison to control counterparts as well as in the expression of pluripotency markers like Oct3/4A, Nanog and Sox2, both at mRNA and protein levels (Figure 1A–C). Expression of these transcription factors, which are the crucial components of self-renewal- and pluripotency-regulating signaling network, additionally indicated successful generation of induced pluripotent stem cells. Both WT and db/db iPSCs presented abundantly the SSEA1 carbohydrate epitopes, bound CDy1 dye (Figure 1C), and expressed alkaline phosphatase (Figure 1D). On the other hand, *Hmox1* (heme oxygenase-1), *Prt4* (proteoglycan-4), and *Agt* (angiotensinogen) showed a tendency toward lower expressions in db/db iPSCs than in WT cells (Figure 1E). This observation seems interesting, as proteoglycan-4 can promote development of hemangioblasts^20^ and similar role has been ascribed to the angiotensin converting enzyme^21^, whereas heme oxygenase-1 is a proangiogenic enzyme necessary for proper function of endothelial progenitors^22^.

**Figure 2:**
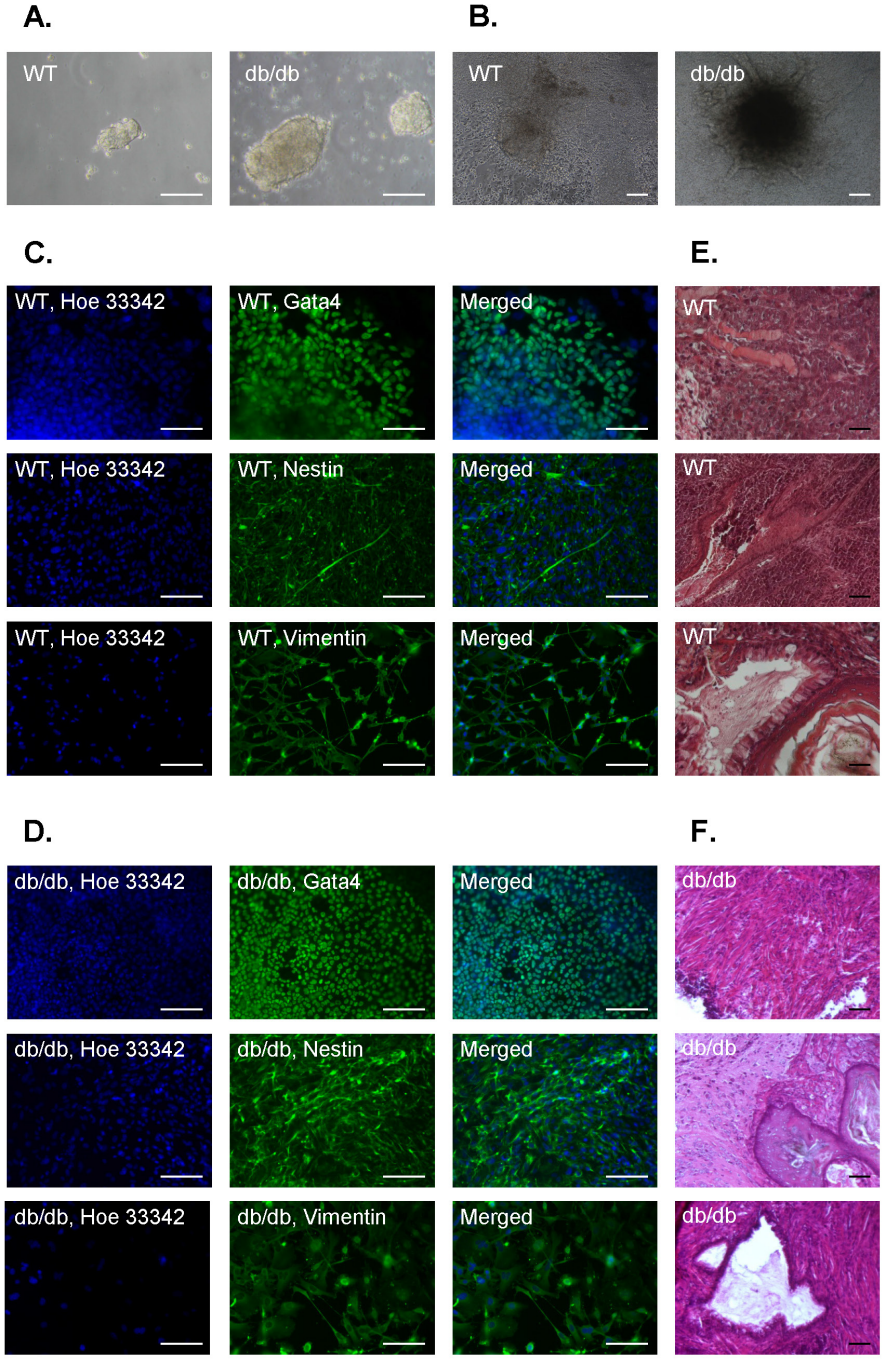
Differentiation potential of wild type (WT) and *Lep^db/db^* (db/db) iPS cells. (A). Embryoid bodies formed from iPS cells after seeding on non-adherent dishes without LIF stimulation (scale bar – 100 μm). (B). Outgrowth of cells from embryoid bodies after adherence to gelatin-coated dishes. Outgrowing cells were further differentiating (scale bar – 100 μm). (C,D). Immunofluorescence staining of markers characteristic for three different germ layers after two weeks of spontaneous differentiation *via* embryoid bodies of WT iPS cells (C) or db/db iPS cells (D). Gata4 – marker of early and defined endoderm as well as mesoderm; nestin – marker of ectoderm; vimentin – marker of mesoderm. Representative pictures (scale bar – 100 μm). (E,F). Teratoma formation assay – representative pictures demonstrating tissues originating from three germ layers within a tumor developed after subcutaneous injection of WT iPS cells (E, N = 3) or db/db iPS cells (F, N = 4) into immunocompromised mice. Upper – muscles (mesodrem), middle – epidermis (ectoderm), bottom – ciliated epithelium (endoderm) (scale bar – 100 μm).

To further verify the pluripotency of iPSCs, they were subjected to spontaneous differentiation process through the formation of embryoid bodies (Figure 2). Indeed, after removal of feeder layer and LIF (leukemia inhibitory factor) stimulation, control and db/db iPSCs generated EBs which adhered to gelatin-coated wells (Figure 2A,B). Importantly, immunofluorescence analysis confirmed the expression of markers of different germ layers in the control and db/db cells that further outgrew from the adhered EBs (Figure 2C,D). Finally, subcutaneous injection of either control or db/db iPSCs into immunocompromised mice resulted in formation of teratomas which were composed of tissues characteristic for endoderm, mesoderm and ectoderm (Figure 2E,F). This experiment proved that both WT iPSCs and those generated from *Lep^db/db^* animals were pluripotent. Additionally, this demonstrated that functional leptin receptor is not crucial for the maintenance of pluripotency in iPSCs.

### Endothelial differentiation of control and db/db iPSCs

**Figure 3:**
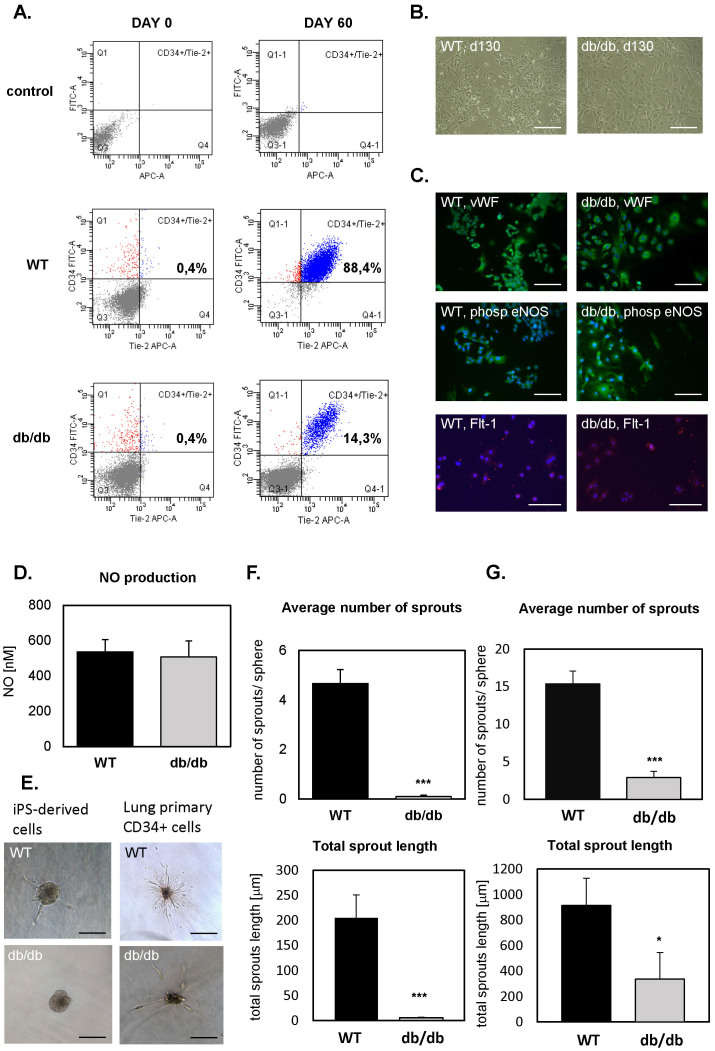
Differentiation of wild type (WT) and *Lep^db/db^* (db/db) iPS cells into endothelial progenitor-like cells. (A). FACS analysis of CD34^+^Tie-2^+^ population in WT and db/db iPSC-derived cells before differentiation (day 0) and after establishing the differentiated cell lines (day 60). Representative dot-plots. Control – unstained cells. (B). Morphology of WT and db/db iPSC-derived endothelial progenitor-like cell lines at 130^th^ day of culture. Representative pictures. (C). Immunofluorescence staining of von Willebrand Factor (vWF), phosphorylated endothelial nitric oxide synthase (phosp eNOS), and vascular endothelial growth factor receptor 1 (Flt-1) in WT and db/db iPSC-derived endothelial progenitor-like cells (scale bar – 100 μm). (D). Nitric oxide production by WT and db/db iPSC-derived endothelial progenitor-like cells. Gas-phase chemiluminescence reaction (n = 3). (E). Sprouting spheres embedded in collagen, generated from WT and db/db iPSC-derived endothelial progenitor-like cells or lung primary CD34^+^ endothelial cells isolated from WT and db/db mice. Representative pictures (scale bar – 100 μm). (F,G). Average number of sprouts and total length of sprouts measured for sprouting spheres embedded in collagen, generated from WT (n = 12 spheres) and *Lep^db/db^* (n = 10 spheres) iPSC-derived endothelial progenitor-like cells (F) or lung primary CD34^+^ endothelial cells isolated from WT (N = 5) and db/db (N = 4) mice (G). Each bar represents mean + SEM. * *p* < 0.05, *** *p* < 0.001.

To stimulate endothelial differentiation, control and db/db iPSCs were seeded on collagen IV to induce differentiation into mesoderm lineages and subsequently cultured on fibronectin in the presence of vascular endothelial growth factor (VEGF) to direct the process toward endothelial cells^23^. Of note, the fraction of CD34^+^Tie-2^+^ subpopulation was much lower within differentiating db/db cells than in control counterparts (14.3% *versus* 88.4% at day 60 of differentiation, Figure 3A), suggesting that functional leptin signaling might influence this process. Nevertheless, either control or db/db cells acquired characteristic endothelial-like cobblestone morphology (Figure 3B), and expressed endothelial markers, such as von Willebrand Factor (vWF), phosphorylated endothelial nitric oxide synthase (eNOS), and vascular endothelial growth factor receptor 1 (Flt-1) (Figure 3C). Phosphorylation of eNOS protein indicated its enzymatic activity. Indeed, the iPSC-derived endothelial progenitor-like cells produced nitric oxide (NO), with the same efficacy for both genotypes (Figure 3D).

Then, we compared the angiogenic potential of the iPSC-derived cells using a capillary sprouting assay (Figure 3E). Cells were suspended in medium containing methyl cellulose and seeded in U-shaped 96-well plate to form spheroids, which were subsequently embedded in collagen and stimulated with VEGF to produce capillary-like sprouts. The number and length of sprouts were analyzed after 48 h, but in WT spheroids the outgrowth of sprouts was visible already after 24 h. In contrast, db/db spheroids formed almost no capillary-like structures (Figure 3E,F). Namely, we demonstrated a significant impairment both in number and length of capillary like structures (Figure 3F). This observation suggests that cells obtained after endothelial differentiation of db/db iPSCs display impaired angiogenic potential, the feature typical for endothelial cells isolated from db/db mice. Indeed, we found a very similar impairment when spheroids were formed by CD34^+^ primary endothelial cells isolated from the lungs of wild type or db/db mice (Figure 3G).

### Generation and characterization of HNF1A MODY reprogrammed cells

**Figure 4:**
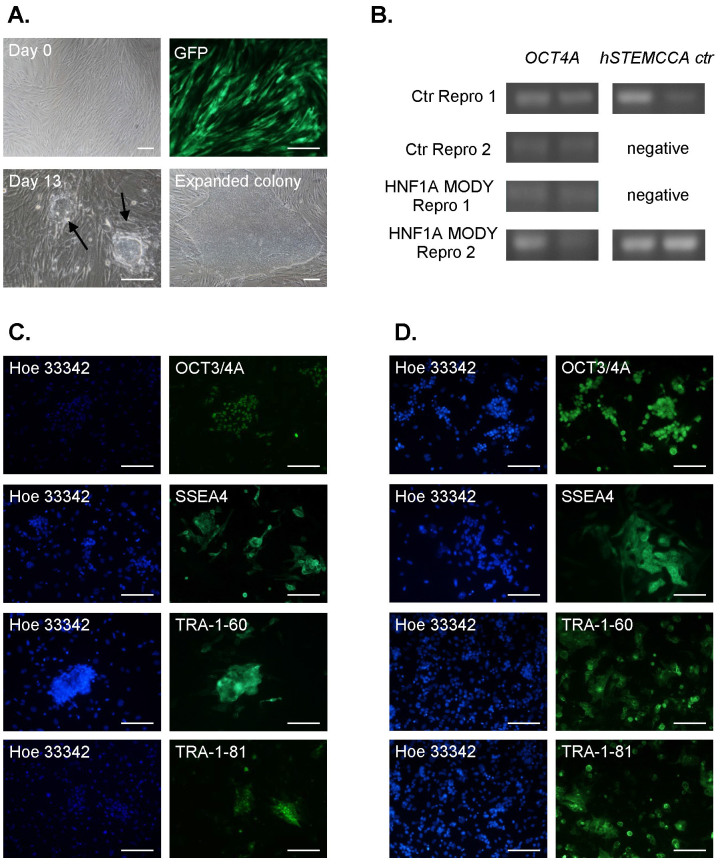
Generation of human reprogrammed cells from fibroblasts of control healthy subjects and HNF1A MODY patients. (A). Morphology of cells from healthy subject before (day 0) and 13 days after transduction (day 13) with hSTEMCCA lentiviral vectors. Colonies resembling human embryonic stem cells, visible at day 13, were further expanded (expanded colony, shown at passage 2). GFP - expression of GFP in human primary fibroblast isolated from HNF1A MODY patients, 72 after transduction with control (FUGW) lentiviral vectors. Representative pictures (scale bar – 100 μm). (B). Analysis of hSTEMCCA construct silencing in established control (effective silencing in Ctr Repro 2 cell line) and HNF1A MODY reprogrammed cells (effective silencing in HNF1A MODY Repro 1 cell line). RT-PCR. (C,D). Immunofluorescence staining of pluripotency markers: OCT3/4A, SSEA4, TRA-1-60, TRA-1-81 in control reprogrammed cells (C) and HNF1A MODY reprogrammed cells (D). Representative pictures (scale bar – 100 μm).

Similarly to mouse tail tip fibroblasts, human primary skin fibroblasts isolated from non-diabetic controls and HNF1A MODY patients were successfully reprogrammed using STEMCCA vectors. Multiple colonies were obtained 3 to 4 weeks after lentiviral transduction (Figure 4A). Since initially we encountered very low efficiency of successful expansion of reprogrammed after picking up and passaging single colonies, a different procedure was tested, where the generated colonies were detached together with non-reprogrammed fibroblasts and seeded onto gelatin-coated wells. After 0.5 h most of the fibroblasts, in contrast to reprogrammed cells, had already attached to the bottom of the well, which enabled a significant enrichment of the floating cell population with reprogrammed cells. Non-adhered cells were collected, seeded on Matrigel and further cultured. This step was helpful in successful expansion of human reprogrammed cells in our hands.

**Figure 5:**
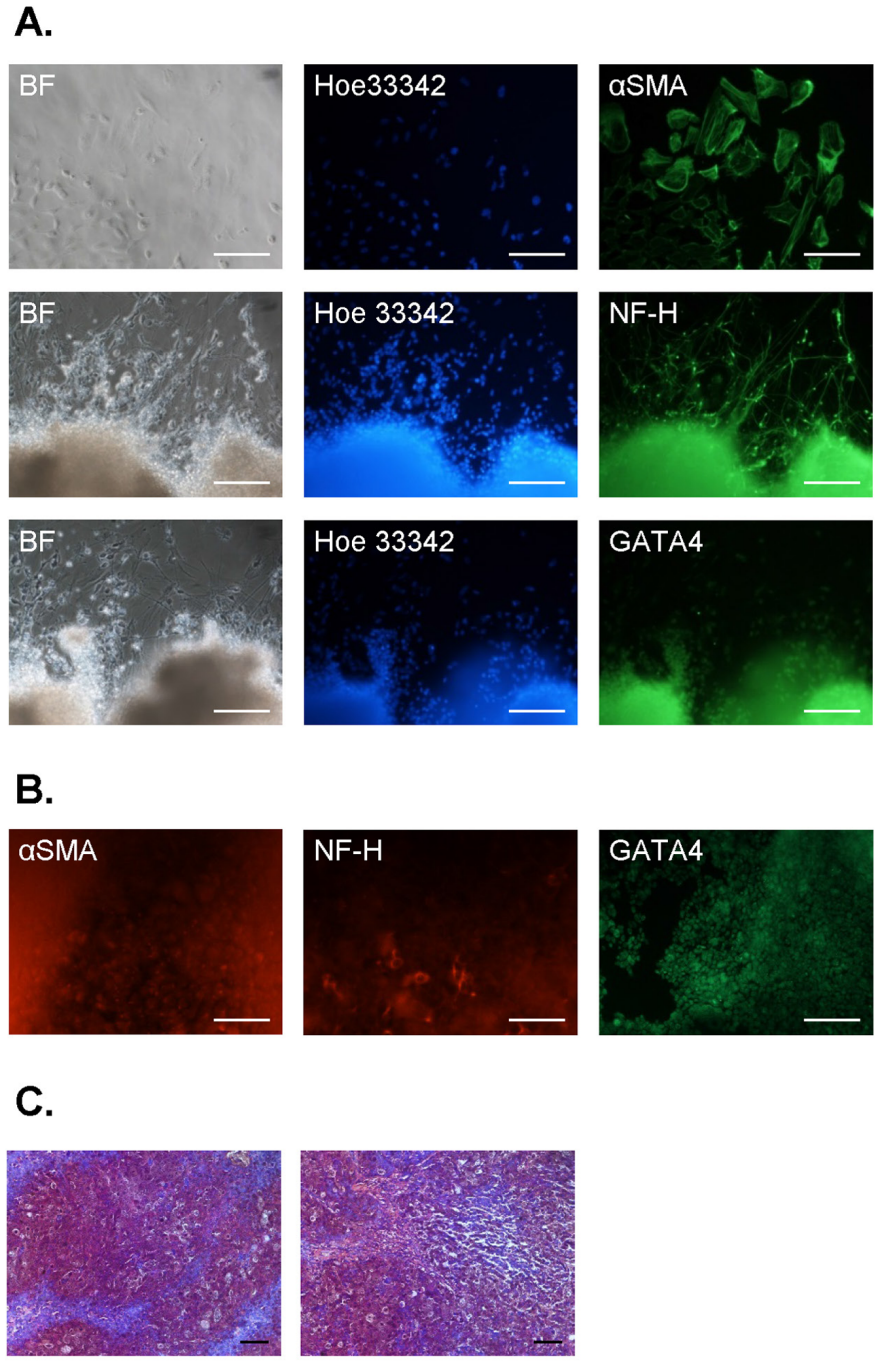
Differentiation potential of human control and HNF1A MODY reprogrammed cells. (A,B). Immunofluorescence staining of markers characteristic for three different germ layers after two weeks of spontaneous differentiation *via* embryoid bodies. A – differentiating control reprogrammed cells. B – differentiating HNF1A MODY reprogrammed cells. GATA4 – marker of early and defined endoderm as well as mesoderm; neurofilament heavy chain (NF-H) – marker of ectoderm; α smooth muscle actin (αSMA) – marker of mesoderm (scale bar – 100 μm). (C). Histological staining of epithelial tumor formed in immunocompromised mouse after injection of human control reprogrammed cells. Representative pictures (scale bar – 100 μm).

Further characterization of the obtained control and HNF1A MODY reprogrammed cells confirmed silencing of lentiviral reprogramming construct in two of four lines (Ctr Repro 2 and HNF1A MODY Repro 1, Figure 4B). Both control and HNF1A MODY cells expressed pluripotency markers, as shown using immunofluorescence analysis of OCT3/4A, SSEA4, TRA-1-60, and TRA-1-81 (Figure 4C,D). Concomitantly, they formed embryoid bodies after removal of bFGF stimulation and seeding on non-adherent dishes (Figure 5A). When EBs were transferred to the adherent, Matrigel-coated wells, the outgrowth of differentiating cells could be detected in the following days (Figure 5A). These cells expressed markers characteristic for all three germ layers, confirming the broad differentiation potential of generated human reprogrammed cells (Figure 5 A,B). Nevertheless, despite multiple cell injections into immunocompromised mice, no teratoma generation was observed. In case of Ctr Repro 2, one of the injections resulted in development of malignant epithelial tumor (Figure 5 C).

### Differentiation of control and HNF1A MODY reprogrammed cells toward a pancreatic lineage

**Figure 6:**
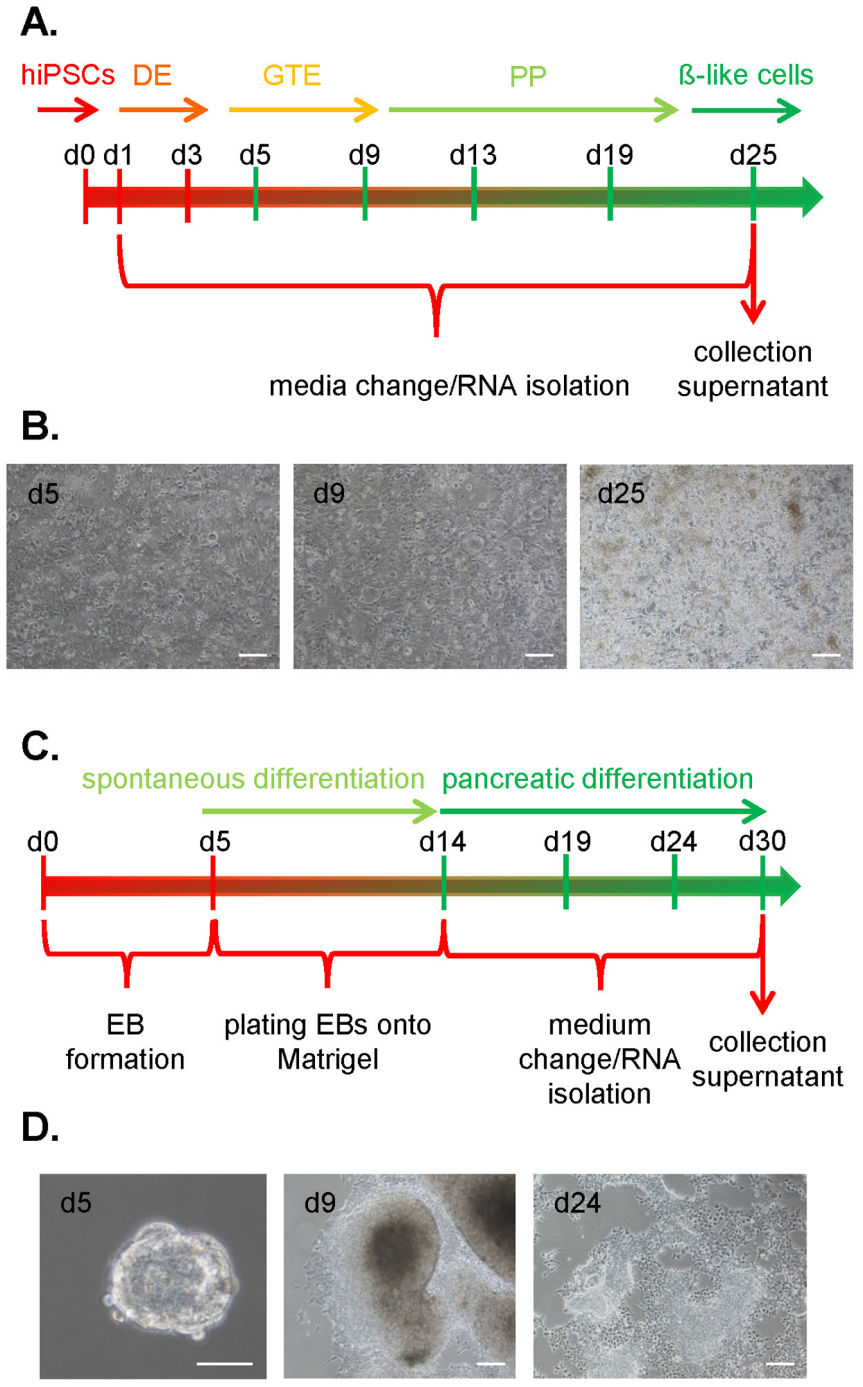
Differentiation of human reprogrammed cells to pancreatic lineage. (A). Schematic representation of direct differentiation method. (B). Morphological changes of HNF1A MODY Repro 1 line during direct differentiation (scale bar – 100 μm). (C). Schematic representation of embryoid body-mediated differentiation method. (D). Morphological changes of HNF1A MODY Repro 1 line during embryoid body-mediated differentiation. Representative pictures (scale bar – 100 μm).

To assess the potential of HNF1A MODY reprogrammed cells to differentiate toward insulin-producing cells, two methods have been tested, namely direct and embryoid bodies-mediated differentiation. The efficacies of these approaches were evaluated by analysing the expression of specific markers at mRNA levels. RT-PCR assay was employed to demonstrate expression of i) *SOX17* and *FOXA2*, markers of definitive endoderm (DE, day 1–3), ii) *HNF4*α, marker of gut endoderm (GTE, d3–9), and iii) *HNF6*, *PDX1*, *HB9*, *NKX6.1* and *NKX2.2*, markers of pancreatic progenitor cells (PP, d9–19). The five latter mentioned markers were analyzed along with expression of insulin (*INS*) in the last step of development toward β-like cells (d19–25). A scheme describing the stages and time points of differentiation as well as photos of differentiating cells are shown in Figure 6 A&B. For the EB-based approach only three time points were tested, namely day 19^th^, 24^th^ and 30^th^ (day 5^th^, 10^th^ and 16^th^ upon seeding of cells on collagen I-coated plates (Figure 6C). To monitor the progression, the same markers as in the direct differentiation were analysed (*SOX17*, *FOXA2, HNF4*α at day 19^th^; *HNF6*, *PDX1*, *HB9* and *NKX6.1* at day 24^th^; *NKX2.2* and insulin after 30 days of differentiation). Morphology of differentiating cells in EB-based method is shown in Figure 6D.

**Figure 7:**
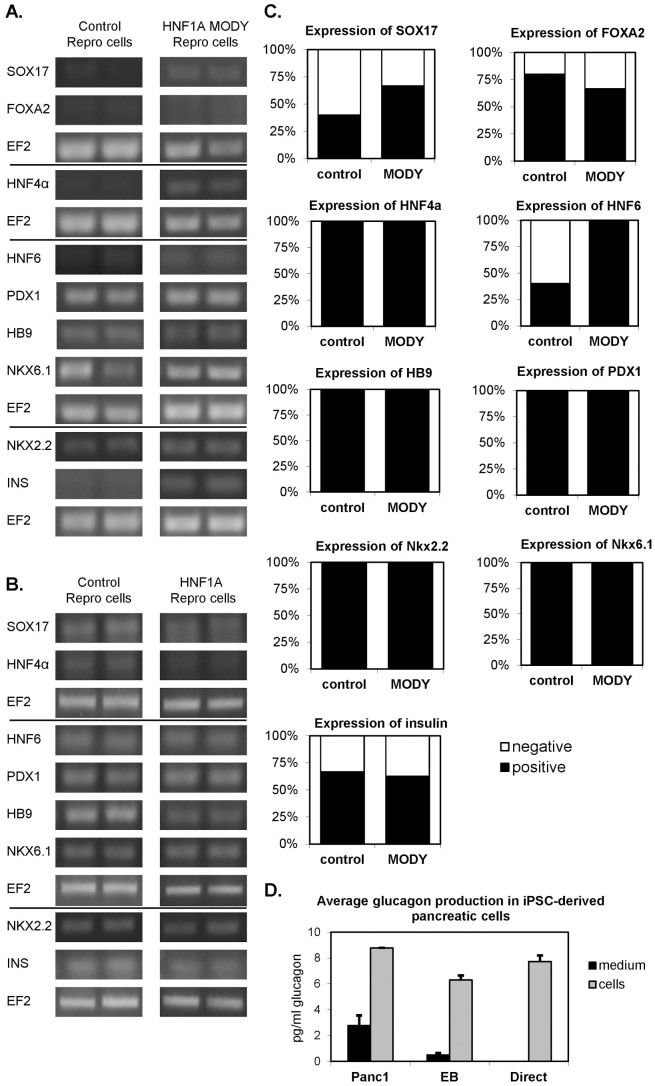
Expression of markers of pancreatic differentiation in cells derived from human control and HNF1A MODY reprogrammed cells. (A). Expression of markers in cells subjected to direct differentiation method. (B). Expression of markers in cells subjected to embryoid bodies-mediated method. Agarose gel electrophoresis of representative RT-PCR products. In case of direct differentiation RT-PCR was performed for cells from each patients and in case of embryoid bodies-mediated differentiation RT-PCR was performed in duplicates. EF2 was used as a housekeeping control for each performed reaction. (C). The efficiency of detection of pancreatic differentiation markers (both differentiation methods are combined, n = 3–5). Percentage of positive RT-PCR results in relation to expression of EF2 housekeeping gene which was detected in all analysed samples. (D). Production of glucagon in differentiated Panc-1 cells as well as control and HNF-1A MODY reprogrammed cells differentiated with embryoid bodies-mediated method (EB) and direct method (direct). Medium – media used to perform the last step of differentiation (in case of Panc-1 cells one type of medium was utilized); cells – media collected from differentiated cells in the last day of differentiation (n = 2).

In case of the direct approach, all specific markers were detectable in differentiating cell lines either derived from control or HNF1A MODY patients (Figure 7A). Similarly, in the method based on EB-mediated differentiation all markers, except *FOXA2*, were detectable in control and HNF1A MODY cells (Figure 7B). It must be stressed, however, that not all RT-PCR analyses produced the positive signals (Fig. 7C). This might result from a low efficiency of differentiation or not optimal time-point chosen for particular cell line. Importantly, markers of pancreatic progenitor cells (*HB9*, *PDX1*, *NKX2.2*, and *NKX6.1*) were detectable in each reaction performed (Fig. 7C). Nevertheless, in all cell lines studied the insulin was expressed at a very low level, what precluded performing reliable quantitative measurements (Fig. 7A–C). Accordingly, we could not reliably detect C-peptide in media collected from differentiated cells, however we were able to measure the concentration of released glucagon (Fig. 7). The presence of glucagon indicates that utilized protocols enabled generation of immature, polyhormonal pancreatic cells, which is a usual outcome of *in vitro* differentiation methods^24^.

## Discussion

We report in this study the successful generation of murine iPSCs from wild type and diabetic db/db animals, as well as reprogrammed human cells from control individuals and diabetic HNF1A MODY patients. Both mouse iPSCs and human cells expressed pluripotency markers like Oct3/4A or Nanog and they were able to differentiate *in vitro* into cells originating from three different germ layers. After injections of mouse control and db/db iPSCs into immunocompromised animals we could observe teratoma formation which indicates that obtained cell lines were pluripotent. Additionally, mouse iPSCs could be differentiated into endothelial progenitor-like cells positive for CD34 and Tie-2 markers. Importantly, such iPSc-derived db/db cells displayed impairment in angiogenic potential similar to that demonstrated by mature primary endothelial cells isolated from lungs of db/db mice. In case of human reprogrammed cells, established control and HNF1A MODY lines did not form teratomas, nonetheless, they could be directed to differentiate toward pancreatic lineage.

To the best of our knowledge this is the first report indicating successful generation of iPSCs from *Lep^db/db^* mice which are the model of T2D. Liu *et al.* previously obtained and characterized iPSCs from NOD mice which are used to study pathophysiology of diabetes type 1^19^. Additionally, in another study such pluripotent cells were successfully differentiated into functional pancreatic beta-like insulin-producing cells which, upon transplantation into diabetic mice, reversed hyperglycemic phenotype of these animals^25^.

Endothelial dysfunction is an important complication associated with T2D^26^. Both hyperglycemia and insulin resistance contribute to impaired physiology of endothelial cells by increasing reactive oxygen species, production of vasoconstrictive factors, and decreasing NO bioavailability^27^. Interestingly, many reports indicated that diabetes negatively affects a heterogeneous population of endothelial (proangiogenic) progenitor cells^28^,^29^, claimed to originate from bone marrow^22^. Obesity and insulin resistance, the hallmarks of T2D, lead to elevated leptin levels which in turn can result in tissue leptin resistance and endothelial dysfunction^30^. Additionally, leptin receptor was reported to mediate the mobilization of bone marrow-derived proangiogenic progenitor cells^31^.

The existence of endothelial progenitors in bone marrow, specific set of markers which should be used for their characterization, and their real contribution to the process of angiogenesis is still disputable^32^, hence better models are required. Moreover, leptin whose signaling pathways exerted through ObR receptor are abrogated in db/db mice, was previously demonstrated to be a proangiogenic factor^33^,^34^ and regulator of vascular contractility^35^,^36^. On the other hand, hyperleptinemia and tissue leptin resistance, being the hallmarks of obese diabetic patient and db/db animals, negatively affect functioning of endothelial cells and constitute important risk factors for the development of cardiovascular disease^30^. Taking this into consideration we decided to evaluate the potential of db/db iPSCs to differentiate towards endothelial cells. We demonstrated here the successful generation of CD34^+^Tie-2^+^ population of progenitor-like cells of endothelial morphology, expressing active endothelial nitric oxide synthase, from both control and db/db iPSCs. Importantly, despite similar expression of endothelial markers, the proangiogenic activities of the db/db iPSC-derived cells were impaired as that of the mature endothelial cells isolated from db/db mice. This suggests that iPSC-derived endothelial-like cells may be useful in investigating the intrinsic cellular mechanisms associated with endothelial dysfunction in diabetes of different genetic background.

Since expression of CD34 is characteristic for progenitor cells^32^, the obtained population of differentiated cells could be considered as enriched in endothelial progenitor-like cells. The presence of Tie-2 protein, the receptor recognized by angiopoietin-1 (Ang1), a potent proangiogenic factor^37^, further confirms their endothelial phenotype. Interestingly, within the iPSCs colonies from which successful generation of CD34^+^Tie-2^+^ populations was achieved we observed lower efficiency of differentiation in db/db cells. This might be in line with the decreased expression of heme oxygenase-1, angiotensinogen and proteoglycan-4 in db/db iPSCs. Among these factors, heme oxygenase-1 is required for proper angiogenic function of mature endothelial cells^38^ and bone marrow-derived proangiogenic cells [ref. 22, reviewed in ref. 39]. The role of angiotensinogen and proteoglycan-4 in endothelial differentiation and physiology has not been extensively studied, however, they can facilitate development of hemangioblasts^20^,^21^.

Differentiated db/db cells exhibited impaired angiogenic potential in endothelial sprouting assay. Schiekofer *et al.* demonstrated that db/db mice have reduced basal capillary density and impaired ischemia-induced vascular remodeling which was associated with altered expression patterns of angiogenesis-related factors in db/db animals^40^. We also showed that proangiogenic cells derived from bone marrow of db/db mice display lower migratory capacity and reduced formation of capillaries both in 2D culture on Matrigel and in 3D culture of spheroids embedded in collagen^41^. Our present results could additionally indicate that leptin plays an important role in embryonic development of endothelial cells, although more studies are necessary to draw reliable conclusions.

In contrast to human T2D, Maturity Onset Diabetes of the Young 3 is a monogenic disease and results from the mutations in HNF-1α transcription factor. Although impaired function of only one protein is linked to the pathophysiology of HNF1A MODY, the molecular mechanisms responsible for the clinical outcome of the patients are not fully understood, mainly due to the poor accessibility of affected tissues. Generation of patient-specific iPSCs could be an important step to address this issue. Indeed, HNF1A MODY iPSCs have been already obtained by Teo *et al.* who utilized Cre-excisible STEMCCA vector^17^. However, the authors did not perform pancreatic differentiation of those cells.

The reprogrammed cells obtained in our work expressed pluripotency markers like OCT3/4A, SSEA4, TRA-1-60 and TRA-1-81 and differentiated *in vitro* towards cells originating from three germ layers. However, despite positive staining for pluripotency and lineage-commitment markers, they were not able to form teratomas after subcutaneous administration into immunocompromised mice. This could indicate that the obtained cell lines represent only partially reprogrammed cells^42^. Alternatively, our method of establishing stable iPSC lines could result in heterogeneous population of partially and fully reprogrammed cells with varying proportions of both populations. In this scenario, the lack of teratoma formation could result from too low number of bona fide iPSCs among injected cells.

Our data indicate that obtained iPSCs could be successfully differentiated into cells of pancreatic lineage, expressing the insulin gene and producing glucagon. Importantly, in view of this differentiation potential, lack of teratoma formation can be considered as beneficial characteristic increasing the safety of possible future clinical utilization of such cells. Utilization of such patient-specific cells to generate cells of pancreatic lineages would provide an excellent tool to study molecular mechanisms responsible for impaired insulin production in HNF1A MODY patients and the exact role of HNF-1α in this process. The proof-of-concept of such approach was provided by Hua *et al.* with iPSCs derived from MODY2 patients who carry the mutations in glucokinase encoding gene^18^. Those cells could be differentiated into mature β cells with similar efficiency as control counterparts and recapitulated the clinical phenotype of the patients. This study highlights also the important issue of utilization of proper control cells which the authors generated by targeted gene modifications with zinc finger nucleases. Such approach enables the direct comparison of mutated and corrected cells with the same genetic background. In our study, the control cells were derived from non-diabetic individuals so the possibility of differences originating from different genetic background of HNF1A MODY reprogrammed cells should be taken into consideration. Nevertheless, the level of expression of insulin gene in our *in vitro* differentiated pancreatic lineage was too low for reliable quantitative comparisons and mechanistic study of molecular pathways. One can suppose that the final steps of differentiation within *in vivo* niche can be more effective^18^

During the revision of this manuscript, Pagliuca *et al.* reported novel *in vitro* differentiation approach leading to generation of functional human pancreatic β cells from pluripotent stem cells^24^. Importantly, such stem-cell-derived glucose-responsive and monohormonal insulin-producing β cells demonstrated features of mature adult counterparts. This is of great importance since previous protocols, including those utilized in this study enabled predominantly to obtain immature, polyhormonal cells^24^,^43^. Accordingly, our differentiation approaches led to generation of cells expressing insulin and glucagon.

In summary we report in this study the generation of mouse db/db iPSCs as well as patient-specific HNF1A MODY reprogrammed cells which could be considered as important tools for investigation of molecular mechanisms responsible for pathophysiology of different types of diabetes. Further studies should elucidate the potential of such cells in modeling clinical phenotypes observed in diabetic patients.

## Methods

### Mice

*Lep^db/db^* and control mice were purchased from Charles River. C57BL/6J mice (Charles River) were used to prepare mouse embryonic fibroblasts constituting a feeder layer for the growth of iPSCs. Foxn1nu immunodeficient mice utilized in teratoma formation assay were purchased from Harlan Laboratories. Mice were maintained under the specific pathogen free conditions, in individually ventilated cages, with full access to food and water. All procedures involving the use of animals were performed according to approved guidelines. All animal experiments were approved by the Local Ethical Committee for Animal Research at the Jagiellonian University (approval no. 51/2012, 101/2013, 208/2013).

### Preparation and culture of murine fibroblasts

Somatic fibroblasts were isolated from tails of 3–4 month old *Lep^db/db^* (N = 5) and control mice (N = 5). Tails were excised, peeled, minced into smaller pieces and subsequently digested in collagenase II solution (1.5 mg/mL, Life Technologies) for 3 hours. Tissue fragments were centrifuged and seeded onto gelatin-coated wells of 6-well plates in DMEM (Lonza) containing 10% FBS (Lonza), penicillin (100 U/mL) and streptomycin (100 μg/mL, both from Sigma-Aldrich). The same medium was used for further culture of the cells.

Mouse embryonic fibroblasts (MEFs) were obtained from 13–15 day old C57BL/6J embryos. After isolation from the uterus, embryos were washed twice in PBS and subjected to the head and visceral tissues removal. The remaining parts were minced and digested in 0.25% trypsin/EDTA solution (1 mL per embryo, Lonza) for 2 hours. Subsequently, fresh DMEM medium containing 10% FBS, penicillin (100 U/mL) and streptomycin (100 μg/mL) was added (5 mL per embryo), obtained cells and tissue fragments were centrifuged (200 × *g*, 5 minutes) and seeded onto 75 cm^2^ flasks. The same type of medium was used for further culture of MEFs. For mitotical inactivation of these cells, mitomycin C (Sigma-Aldrich) was added to the culture medium at the concentration of 10 μg/mL for 3 hours. Subsequently, inactivated MEFs (iMEF) were seeded to constitute a feeder layer for growth of either mouse iPSCs (1.5 × 10^5^ cells/well of 6-well plate) or human (3 × 10^5^ cells/well of 6-well plate) reprogrammed cells.

### Patient selection, skin biopsies and culture of human primary fibroblasts

All procedures have been performed according to the approved guidelines of Local Bioethical Committee at the Jagiellonian University, after receiving the informed consent of all the subjects (Local Bioethical Committee's approval no. KBET/43/B/2012). Patients suffering from HNF1A MODY were recruited in the Clinic of Metabolic Disease, Collegium Medicum of Jagiellonian University, Krakow. HNF1A MODY Repro 1 cell line was derived from 57 years old female with substitution of cytosine 811 with thymidine within exon 4 of *HNF1A* gene leading to replacement of arginine with tryptophan in protein sequence. HNF1A MODY Repro 2 cell line was derived from 30 years old male with deletion of thymidine 1137 within exon 6 of *HNF1A* gene leading to the shift in protein coding frame. 6 mm skin biopsies were transferred into PBS (Mg^2+^ and Ca^2+^ free) supplemented with penicillin (100 U/mL), streptomycin (100 μg/mL) and amphotericin B (10 μg/mL, Sigma-Aldrich). Dermis was subsequently separated from epidermis and cut into small pieces which were digested in collagenase II (2.75 mg/mL) and DNaseI (1 mg/mL, Merck) solution. Released cells and tissue fragments were then centrifuged (200 × *g*, 5 minutes), suspended in fresh DMEM medium containing 10% FBS, penicillin (100 U/mL), streptomycin (100 μg/mL) and amphotericin B (10 μg/mL) and seeded on a gelatin-coated wells of 6-well plate. The same medium was used for further culture of human fibroblasts.

Control, non-diabetic samples were obtained in the Clinic of Surgery, Collegium Medicum of Jagiellonian University, Krakow, from two non-diabetic patients undergoing routine surgery. For this purpose, surgical incisions were prolonged for 4–6 mm, and skin biopsies were collected. Further isolation of human fibroblasts was performed following the same procedures as MODY samples.

### Production of VSV-G pseudotyped lentiviral vectors

Production of VSV-G pseudotyped lentiviral vectors was performed according to the previously described protocol^44^. Briefly, 7 × 10^6^ 293 T cells (kindly gifted by dr. Maciej Wiznerowicz, Greater Poland Cancer Center, Poznan, Poland) were seeded onto 10 cm dishes in the DMEM medium containing 10% FBS, penicillin (100 U/mL) and streptomycin (100 μg/mL) and subsequently subjected to the triple plasmid transfection with polyethylenimine (PEI, Polysciences Inc.) as a transfectant reagent. Reprogramming vectors were produced utilizing hSTEMCCA plasmid construct containing four transcription factors: OCT3/4A, KLF4, SOX2 and c-MYC within one expression cassette (kindly provided by dr. Gustavo Mostoslavsky, Boston University, USA^45^) whereas in case of control vectors, FUGW plasmid, harboring GFP cDNA, was used (Addgene 14883). Additionally, psPAX2 and pMD2.G plasmids (kindly provided by dr Maciej Wiznerowicz, Greater Poland Cancer Center) served as a source of tat, rev, gag/pol and VSV-G, respectively. 48 h post-transfection, media containing vectors were collected, filtered through 0.8 μm filters (Millipore), aliquoted, and frozen at −80°C.

### Reprogramming of mouse and human fibroblasts

2.5 × 10^4^ murine fibroblasts isolated from wild type as well as *Lep^db/db^* mice were seeded on wells of 24-well plate and transduced with either reprogramming (hSTEMMCA) or control, FUGW vectors, in the presence of 4 μg/mL of polybrene (Sigma-Aldrich). 24 h post-transduction media containing vector particles were replaced with fresh culture media, in which the cells were grown until full confluency. Subsequently, fibroblasts were trypsinized (0.25% trypsin/EDTA solution) and transferred to the 6-well plates covered with mitotically inactivated MEFs in the iPSCs medium consisting of DMEM supplemented with 20% FBS, penicillin (100 U/mL), streptomycin (100 μg/mL), 1% non-essential amino acids (Life Technologies), 0.1 mM 2-mercaptoethanol (Life Technologies) and 1000 U/ml leukemia inhibitory factor (LIF, Millipore). The medium has been changed daily until the colonies resembling embryonic stem cells emerged. Colonies were subsequently picked, trypsinized (0.25% trypsin/EDTA solution) and seeded on the new wells of 24-well plate coated with mitotically inactivated MEFs. Control and *Lep^db/db^* iPSCs were further expanded and characterized.

For reprogramming of human fibroblasts, 2.5 × 10^4^ control and HNF1A MODY cells were seeded on gelatin-coated (Sigma-Aldrich) 24-well plates and transduced with either reprogramming (hSTEMMCA) or control, FUGW vector in the presence of 4 μg/mL of polybrene (Sigma-Aldrich). 24 h post-transduction media containing vector particles were replaced with human iPSC medium consisting of KnockOut DMEM (Life Technologies) supplemented with 20% KnockOut Serum Replacement (KSR, Life Technologies), penicillin (100 U/mL), streptomycin (100 μg/mL), 1% non-essential amino acids, 0.1 mM 2-mercaptoethanol and 10 ng/ml of human bFGF (PeproTech). On 6^th^ day of reprogramming the cells were reseeded on either mitotically inactivated MEF- or Matrigel- (BD Biosciences) coated 60 mm dishes. The cells were fed every other day until mature colonies resembling human embryonic stem cells emerged.

Picked colonies were mechanically dissociated and seeded onto new Matrigel- or iMEF-coated 24-well plates. For the improvement of survival the ROCK inhibitor (10 μM, Abcam) was added to the iPSC culture medium^46^. The separation of reprogrammed cells was also carried out by exploiting faster adherence of non-reprogrammed fibroblasts to gelatin-coated surface. Briefly, cell suspension, dissociated by Accutase (GE Healthcare) was placed on gelatin coated dishes for 35 minutes. Floating cells enriched in human reprogrammed cells were further cultured on Matrigel-coated dishes. This procedure was repeated 3–4 times to ensure sufficient removal of non-reprogrammed cells. Reprogrammed lines from two control individuals (named Ctr Repro 1 and 2) and two HNF1A MODY patients (named HNF1A MODY Repro 1 and 2) were subsequently expanded and characterized.

### Spontaneous differentiation of mouse iPSCs and human reprogrammed cells - embryoid body formation

To verify the pluripotency of obtained mouse iPSCs and human reprogrammed cells, in a first step *in vitro* spontaneous differentiation was performed. Briefly, mouse iPSCs and human reprogrammed cells growing on iMEF-coated wells suspended in either mouse iPSC differentiation medium (DMEM supplemented with 20% FBS, penicillin (100 U/mL), streptomycin (100 μg/mL), 1% non-essential amino acids and 0.1 mM 2-mercaptoethanol) or human iPSC differentiation medium (KnockOut DMEM supplemented with 20% KnockOut Serum Replacement, penicillin (100 U/mL), streptomycin (100 μg/mL), 1% non-essential amino acids and 0.1 mM 2-mercaptoethanol) and seeded on non-adherent 100 mm dishes. After 5 days, mouse EBs were transferred onto gelatin-coated 48-well plates and further cultured for 12 days with daily medium change. Similarly, human EBs were transferred after 5–8 days on Matrigel-coated dishes and cultured for 5–9 days. Both mouse and human differentiated cells were subsequently immunofluorescently analyzed for expression of markers characteristic for cells originating from endo-, ecto- and mesoderm.

### Teratoma formation assay

Mouse iPSCs were trypsinized and reseeded on gelatin-coated wells for 1 h to deplete the population of iMEFs. Floating cells were collected, centrifuged and resuspended in DMEM supplemented with 10% FBS, penicillin (100 U/mL), and streptomycin (100 μg/mL). 1.5 × 10^6^ cells were injected subcutaneously into dorsal flank of immunocompromised Fox1nu mice. After approximately 8 weeks tumors were excised, embedded in paraffin and subjected to histological analysis with hematoxilin and eosin staining.

Human reprogrammed cells were detached with Accutase, centrifuged and resuspended in human iPSCs differentiation medium. 1–2 × 10^6^ cells were injected subcutaneously into dorsal flank of immunocompromised Fox1nu mice. After approximately 8 weeks the sole resulting tumor were excised, embedded in paraffin and subjected to histological analysis with hematoxilin and eosin staining.

### Differentiation of mouse iPSCs into endothelial cells

Mouse iPSCs were trypsinized and reseeded on gelatin-coated wells for 1 h to deplete the population of iMEFs. Floating cells were collected, centrifuged, resuspended in α-MEM (Lonza) supplemented with 10% FBS, penicillin (100 U/mL), streptomycin (100 μg/mL), 1% non-essential amino acids and 0.1 mM 2-mercaptoethanol (EDM, endothelial differentiation medium), and subsequently seeded on 12-well plates coated with collagen IV (Sigma-Aldrich; 5 μg/cm^2^, diluted in 50 mM hydrochloric acid). Cells were cultured for 4 days and then transferred to 12-well plates coated with fibronectin (Sigma-Aldrich; 20 μg/mL, 15–30 minutes at 37°C) in EDM medium supplemented with 50 ng/mL VEGF (Sigma-Aldrich). Further passaging was performed whenever cells reached around 90% confluency. After approximately 30–60 days, when majority of cells acquired endothelial-like phenotype (CD34^+^Tie-2^+33^), the medium was changed to EGM-2MV (Lonza) and the cells were further cultured for the immunophenotyping and functional assays. Differentiated cells could be kept in culture for at least 130 days without changes in the morphology, phenotype or growth capacity.

### Isolation of lung primary endothelial cells

Lungs from 5 control, wild type and 6 *Lep^db/db^* mice were cut in small pieces and digested in 1 U/mL of dispase for 1 h at 37°C with agitation from time to time. The resulting cell suspension was filtered and stained with anti-CD34-FITC conjugated antibody (BD Bioscience). Subsequently cells were incubated with anti-FITC magnetic beads (Myltenyi Biotech) for 15 minutes and separated on AutoMACS (Myltenyi Biotech). CD34 positive cells were then counted and seeded in EGM-2MV with methylcellulose in U-shaped 96-well plate for *in vitro* endothelial sprouting assay. No spheres could be obtained from 2 *Lep^db/db^* mice. In the rest of the sorted cells compact spheres could be formed after 5 days in culture. The resulting spheres were embedded in collagen I and the sprouting was assessed after 24 h.

### Flow cytometry

In order to immunophenotype the cells during endothelial differentiation, they were detached with Accutase (Cytogen), washed with PBS and resuspended in PBS with 2% FBS. Subsequently, the cells were incubated with FcR blocking reagent (Miltenyi Biotech) for 10 minutes at 4°C followed by addition of specific antibodies conjugated with fluorochromes: CD34-FITC (BD Biosciences) and Tie-2-APC (Biolegend). After 30 minutes of incubation at 4°C the cells were washed with PBS, resuspended in PBS supplemented with 2% FBS and acquired on LSRII flow cytometer (BD Biosciences). Appropriate single stained samples were used for compensation of different fluorochromes. Results were analyzed by Diva software (BD Biosciences).

### Endothelial sprouting assay

To form endothelial spheres, differentiating iPSCs at day 120 of culture were detached with Accutase and transferred to 10 mL of medium containing methyl cellulose [composed of 10 mL of methyl cellulose (Sigma-Aldrich; 100%) and 40 mL of EGM-2MV medium] to obtain concentration of 7500 cells/mL. Subsequently, 750 cells/well were seeded on U-shaped 96-well plate in 100 μl of medium. The plate was incubated at 37°C and the formation of spheres was assessed after 1–3 days.

After the spheres were formed they were embedded in collagen type I (BD Biosciences) for evaluation of sprouting capability. Briefly, the spheres were centrifuged (45 × *g*, 3 minutes, room temperature) and resuspended in 500 μL of methyl cellulose supplemented with 30% FBS. Subsequently, 500 μL of HBSS containing phenol red (Sigma-Aldrich) were mixed with 4 mL of 100% collagen I solution followed with its neutralization with sterile 200 mM NaOH. 500 μL of collagen solution was mixed with 500 μL of sphere suspension and immediately poured on 24-well plate. After 15 minutes of incubation at 37°C, the wells were covered with 200 μL of EGM-2MV medium. The sprouting was assessed after 24 h and 48 h using an inverted microscope.

### Nitric oxide production

The production of NO was measured in the culture medium of differentiated cells at day 130 of culture, using gas-phase chemiluminescence reaction. Nitrite, the major oxidation product of NO in the absence of oxyhemoglobin or superoxide anion, is formed when NO reacts with dissolved oxygen. Therefore the nitrite formed in the medium was reduced in a solution of acetic acid in the presence of iodide^47^ and the measurement was performed with 280i Nitric Oxide Analyzer (NOA^TM^, Sievers) equipped with high sensitivity detector for NO.

### Pancreatic differentiation - direct method

The direct differentiation approach follows a modified protocol by Maehr *et al*.^48^. Accordingly, human reprogrammed cells (Ctr Repro 1 and 2 as well as HNF1A MODY Repro 1 and 2) were seeded onto Matrigel coated 12-well plates. The cells were cultured in human iPSCs medium until 80% confluency was observed followed by the first change to differentiation medium. During the four differentiation stages the differentiation medium was replaced six times: at day 1^st^ it composed of Advanced RPMI (Life Technologies) supplemented with Wnt3a (25 ng/mL, Sigma-Aldrich), Activin A (100 ng/mL, PeproTech), 1% Glutamax (Life Technologies), penicillin (100 U/mL) and streptomycin (100 μg/mL); on day 3^rd^ it composed of Advanced RPMI supplemented with 0.2% FBS, Activin A (100 ng/mL), 1% Glutamax, penicillin (100 U/mL) and streptomycin (100 μg/mL); on day 5^th^ it composed of Advanced RPMI supplemented with 2% FBS, FGF10 (50 ng/mL, PeproTech), 25 μM KAAD-Cyclopamine (Sigma-Aldrich), 1% Glutamax, penicillin (100 U/mL) and streptomycin (100 μg/mL), on day 9^th^ it composed of DMEM supplemented with FGF10 (50 ng/mL), 25 μM KAAD-Cyclopamine, 2 μM retinoic acid (Sigma-Aldrich), B27 supplement (1x, Life Technologies), 1% Glutamax, penicillin (100 U/mL) and streptomycin (100 μg/mL); on day 13^th^ it composed of DMEM supplemented with FGF10 (50 ng/mL), 300 nM Indolactam V (Sigma-Aldrich), B27 supplement (1x, Life Technologies), 1% Glutamax, penicillin (100 U/mL) and streptomycin (100 μg/mL); and on day 19^th^ it composed of DMEM supplemented with 10 μM DAPT (Sigma-Aldrich), B27 supplement (1x), 1% Glutamax, penicillin (100 U/mL) and streptomycin (100 μg/mL). With every step the morphological changes were documented and RNA was isolated.

### Pancreatic differentiation – embryoid body-based method

The differentiation approach based on a prior EBs formation was performed according to the modified protocol described in Schroeder *et al.*^49^. Ctr Repro 2 and HNF1A MODY Repro 1 were used in this experiment. EBs were formed as described for spontaneous differentiation of human reprogrammed cells and cultured in non-adherent conditions for 5 days. Subsequently they were seeded onto Matrigel-coated dishes (60 mm) for further 9 days in the same medium during which spontaneous differentiation occurred. On day 14^th^ the cells were washed 2 times with PBS, trypsinized and removed from the dish surface with a cell scraper. Collected cells were suspended in differentiation medium composed of DMEM/F12 (Lonza) supplemented with 10% FBS, 100 μM putrescine (Sigma-Aldrich), 20 nM progesterone (Sigma-Aldrich), laminin (1 μg/mL, Sigma-Aldrich), insulin (25 μg/mL, Sigma-Aldrich), 30 nM sodium selenite (Sigma-Aldrich), transferrin (50 μg/mL, Sigma-Aldrich), B27 supplement (1x), penicillin (100 U/mL) and streptomycin (100 μg/mL) and seeded onto collagen I coated dishes (1:70 in 0.2 N acetic acid). Differentiation medium was changed every other day and RNA isolation was performed on day 19, 24 and 30.

### Differentiation of Panc-1 pancreatic cancer cell line

Panc-1 cells (ATCC, CRL-1469) were cultured in DMEM medium containing 10% FBS, penicillin (100 U/mL) and streptomycin (100 μg/mL) and differentiated toward insulin- and glucagon-expressing cells according to Suzuki J *et al.* protocol^50^. Briefly, 1 × 10^6^ cells cultured on 6-well plate were washed with PBS and treated for 30 s with 0.05% trypsin/EDTA solution at room temperature. Trypsin was removed, cells were washed three times with PBS and subsequently cultured for 6 days in DMEM/F12 medium containing 17.5 mM glucose and 1% BSA. Media were collected and subjected to enzyme immunoassay (EIA) for measurement of glucagon concentration.

### Enzyme immunoassay (EIA)

EIA assay (Sigma-Aldrich) for measurement of glucagon concentration was performed according to manufacturer's protocol. Media collected from differentiated Panc-1 cells and from the last day of differentiation of control and HNF1A MODY reprogrammed cells via direct and EB-mediated protocols were used.

### Isolation of RNA, RT-PCR and real-time qPCR

Total cellular RNA was isolated utilizing the phenol/chloroform extraction. To ensure complete removal of genomic DNA contamination samples were treated with DNase I (Life Technologies). Reverse transcription was performed with M-MuLV Reverse Transcriptase (Thermo Scientific) and oligo(dT) primers (Promega). PCR reaction was performed using Taq polymerase (Promega) under following conditions: 95°C for 5 minutes, 40 cycles of 95°C for 30 seconds, annealing temperature for 60 seconds and 72°C for 45 seconds, with final elongation at 72°C for 5 minutes. The PCR products electrophoresis was performed in 2% agarose gel. Quantitative real-time PCR with melt curve analysis of amplified products was performed with StepOne Plus cycler (Applied Biosystems) and SYBR® Green JumpStart™ Taq ReadyMix™ (Sigma-Aldrich).

In murine iPSCs the expression of *Oct3/4A*, *Nanog*, *Sox2*, *Hmox1*, *Prg4* and *Agt* was determined by quantitative RT-PCR, while in human reprogrammed cells *OCT3/4A* and markers of pancreatic differentiation were checked^48^. The applied PCR program for detection of pancreatic differentiation markers followed the same setting as for *OCT3/4A* except for the annealing temperature which was reduced to 54°C and additionally, 6 μL (instead of 2 μL) of cDNA samples were used. After first examinations the markers with low gene expression, i.e. *SOX17* and *INS* were also amplified by PCR using a highly sensitive DNA polymerase (Q5®High-Fidelity DNA Polymerase [2000 U/mL], New England Biolabs). In case of direct differentiation RT-PCR was performed for cells from each patients and in case of embryoid bodies-mediated differentiation RT-PCR was performed in duplicates. Additionally, silencing of the hSTEMCCA vector was tested by amplification of sequence specific for transgene cassette with primers designed to recognize c-MYC (forward primer) and WPRE (reverse primer) part of the vector construct within the cellular transcripts^51^. EF2 was used as the housekeeping gene. The sequences of primers used in the study are provided in Table 1.

### Immunofluorescent analysis

Mouse iPSCs and human reprogrammed cells as well as differentiating cells growing in 48-well plates were washed with PBS, fixed with 4% paraformaldehyde for 10 minutes in room temperature and permeabilized with 0.1% Triton-X100 in PBS (10 minutes, room temperature). Cells were washed three times with PBS, blocked for 1 h in room temperature with 4% BSA in PBS solution and incubated with the primary antibodies overnight at 4°C. The following primary antibodies were used: Oct3/4A (1:200, Santa Cruz Biotechnology), Nanog (1:200, Abcam), SSEA-1 (1:200, Millipore), SSEA-4, TRA-1-60, TRA-1-81 (1:100, Millipore), Nestin, Vimentin (1:200, Abcam), Neurofilament heavy chain (1:1000, Abcam), smooth muscle actin (1:100, Abcam), GATA-4 (1:50 for human and 1:200 for murine cells, Santa Cruz Biotechnology), Nkx2.5 (1:200, Santa Cruz Biotechnology), vWF (1:100, Abcam), Flt-1 (1:100, Abcam), phospho eNOS (1:100, Abcam).

Subsequently, cells were washed 5 times with PBS and incubated with secondary antibodies (AlexaFluor, Life Technologies) for 1 h in room temperature. This was followed by incubation with 1 μg/mL of Hoechst 33342 in PBS for 10 minutes in room temperature and 4 washing steps with PBS. Cells were eventually visualized under fluorescent microscope (Nikon Eclipse TS100). Additionally, mouse iPSCs were stained with CDy1 (kindly provided by dr Young-Tae Chang, Department of Chemistry, National University of Singapore), a fluorescent dye which selectively marks mouse and human pluripotent stem cells^52^. For this purpose, iPSCs were incubated with 0.1 μg/mL of CDy1 diluted in iPSCs culture medium for 1 h at 37°C. Cells were than washed with PBS and further cultured for 2 h in fresh medium. Colonies were visualized under fluorescent microscope.

### Statistical analysis

Data are presented as mean + SEM. Unpaired two-tailed t-test or exact Fisher test were used to analyze statistical significance. Results were considered as significant at *p* < 0.05.

## Author Contributions

J.D., A.J., J.S., N.K.T. and M. Malecki designed experiments. J.S., D.O., N.K.T. and M.B. performed experiments. M.S. collected patients and healthy donors. M. Matlok performed skin biopsies, G. Dyduch analyzed the tumors, J.S., A.J. and J.D. analyzed results and wrote the manuscript.

## Figures and Tables

**Table 1:** Primers used in the study

Gene	Species	Sequence	Product length
*Oct3/4A*	mouse	CCCCAATGCCGTGAAGTTGGAGAAGGT	452 bp
		TCTCTAGCCCAAGCTGATTGGCGATGTG	
*Nanog*	mouse	CGTTCCCAGAATTCGATGCTT	102 bp
		TTTTCAGAAATCCCTTCCCTCG	
*Sox2*	mouse	TGGGAGGAAGAGGTAACCACG	116 bp
		ACCTACAGCATGTCCTACTCG	
*Hmox1*	mouse	GTGGAGACGCTTTACGTAGTGC	250 bp
		CTTTCAGAAGGGTCAGGTGTCC	
*Agt*	mouse	CCACTGGAGGGGGTCAGTACA	125 bp
		GAGATGCTGTTGTCCACCCAGA	
*Prg4*	mouse	CGTTGCATCCGAGAACCATG	118 bp
		CATCTCCCTGCACAGCTTGA	
*OCT3/4A*	human	CGGAGCCCTGCACCGTCA	221 bp
		GCAGATGGTCGTTTGGCTGAAT	
*hSTEMCCA_ctr*		AGGAGCAAAAGCTCATTTCTG	326 bp
		TCAGCAAACACAGTGCACACC	
*SOX17*	human	AAGGGCGAGTCCCGTATCC	142 bp
		TCAGCGCCTTCCACGACTTGC	
*FOXA2*	human	ATTGCTGGTCGTTTGTTGTG	187 bp
		TACGTGTTCATGCCGTTCAT	
*HNF4α*	human	CATGGCCAAGATTGACAACCT	113 bp
		TTCCCATATGTTCCTGCATCAG	
*HNF6*	human	CGCTCCGCTTAGCAGCAT	61 bp
		GTGTTGCCTCTATCCTTCCCAT	
*MNX1 (HB9)*	human	TCCACCGCGGGCATGATCCT	165 bp
		GCGCTTGGGCCGCGACAGCTA	
*PDX1*	human	CCTTTCCCATGGATGAAGTC	145 bp
		CGAACTCCTTCTCCAGCTCTA	
*NKX6-1*	human	TCAACAGCTGCGTGATTTTC	126 bp
		CCAAGAAGAAGCAGGACTCG	
*NKX2-1*	human	ATGTAAACGTTCTGACAACT	228 bp
		TTCCATATTTGAGAAATGTTTGC	
*INS*	human	GCTGGTACAGCATTGTTCCAC	154 bp
		CTTCTTCTACACACCCAAGACC	
*EF2*	mouse human	GACATCACCAAGGGTGTGCAG	214 bp
		TCAGCACACTGGCATAGAGGC	
